# Clinically node negative breast cancer patients undergoing breast conserving therapy, sentinel lymph node procedure versus follow-up: a Dutch randomized controlled multicentre trial (BOOG 2013-08)

**DOI:** 10.1186/s12885-017-3443-x

**Published:** 2017-07-01

**Authors:** L. M. van Roozendaal, M. L. G. Vane, T. van Dalen, J. A. van der Hage, L. J. A. Strobbe, L. J. Boersma, S. C. Linn, M. B. I. Lobbes, P. M. P. Poortmans, V. C. G. Tjan-Heijnen, K. K. B. T. Van de Vijver, J. de Vries, A. H. Westenberg, A. G. H. Kessels, J. H. W. de Wilt, M. L. Smidt

**Affiliations:** 1 Division of Surgical Oncology, Maastricht University Medical Centre, P.O. Box 5800, 6202 AZ Maastricht, The Netherlands; 2 GROW - School for Oncology and Developmental Biology, Maastricht University Medical Centre, Maastricht, the Netherlands; 3 Division of Surgical Oncology, Diakonessenhuis Hospital, Utrecht, the Netherlands; 4 Division of Surgical Oncology, Netherlands Cancer Institute - Antoni van Leeuwenhoek Hospital, Amsterdam, the Netherlands; 5 Division of Surgical Oncology, Canisius-Wilhelmina Hospital, Nijmegen, the Netherlands; 6 Department of Radiation Oncology, Maastricht University Medical Centre (MAASTRO clinic), Maastricht, the Netherlands; 7 Division of Medical Oncology, Netherlands Cancer Institute - Antoni van Leeuwenhoek Hospital, Amsterdam, the Netherlands; 8 Department of Radiology and Nuclear Medicine, Maastricht University Medical Centre, Maastricht, the Netherlands; 9 Department of Radiation Oncology, Radboud University Medical Centre, Nijmegen, the Netherlands; 10 Division of Medical Oncology, Maastricht University Medical Centre, Maastricht, the Netherlands; 11 Department of Pathology, Netherlands Cancer Institute - Antoni van Leeuwenhoek Hospital, Amsterdam, the Netherlands; 12 Department of Medical and Clinical Psychology, Tilburg University, Tilburg, the Netherlands; 13 Radiation Oncology, Radiotherapy group, Arnhem, the Netherlands; 14 Department of Clinical Epidemiology and Medical Technology Assessment, Maastricht University Medical Centre, Maastricht, the Netherlands; 15 Division of Surgical Oncology, Radboud University Medical Centre, Nijmegen, the Netherlands

**Keywords:** Breast neoplasms, Breast cancer, Sentinel lymph node biopsy, Breast conserving therapy

## Abstract

**Background:**

Studies showed that axillary lymph node dissection can be safely omitted in presence of positive sentinel lymph node(s) in breast cancer patients treated with breast conserving therapy. Since the outcome of the sentinel lymph node biopsy has no clinical consequence, the value of the procedure itself is being questioned. The aim of the BOOG 2013–08 trial is to investigate whether the sentinel lymph node biopsy can be safely omitted in clinically node negative breast cancer patients treated with breast conserving therapy.

**Methods:**

The BOOG 2013–08 is a Dutch prospective non-inferiority randomized multicentre trial. Women with pathologically confirmed clinically node negative T1–2 invasive breast cancer undergoing breast conserving therapy will be randomized for sentinel lymph node biopsy versus no sentinel lymph node biopsy. Endpoints include regional recurrence after 5 (primary endpoint) and 10 years of follow-up, distant-disease free and overall survival, quality of life, morbidity and cost-effectiveness. Previous data indicate a 5-year regional recurrence free survival rate of 99% for the control arm and 96% for the study arm. In combination with a non-inferiority limit of 5% and probability of 0.8, this result in a sample size of 1.644 patients including a lost to follow-up rate of 10%. Primary and secondary endpoints will be reported after 5 and 10 years of follow-up.

**Discussion:**

If the sentinel lymph node biopsy can be safely omitted in clinically node negative breast cancer patients undergoing breast conserving therapy, this study will cost-effectively lead to a decreased axillary morbidity rate and thereby improved quality of life with non-inferior regional control, distant-disease free survival and overall survival.

**Trial registration:**

The BOOG 2013–08 study is registered in ClinicalTrials.gov since October 20, 2014, Identifier: NCT02271828. https://clinicaltrials.gov/ct2/show/NCT02271828

## Background

More than fifteen years ago, the sentinel lymph node biopsy (SLNB) was introduced in clinically node negative breast cancer patients to evaluate their lymph node status for diagnostic purposes. In case of a negative sentinel lymph node (SLN) an axillary lymph node dissection (ALND) was omitted. The SLN is negative in approximately 74% of patients in a general breast cancer population [1, 2]. Although SLNB is less invasive compared to ALND, short-term complications still occur in 25% of the patients. Most reported complications are axillary seroma, wound infections, hematoma, anaphylactic reaction, axillary paresthesia, and lymphedema, which is described in 8% of patients after a follow-up of only 3 years, resulting in significant reduction of quality of life (QoL) of breast cancer survivors [3–7].

Ever since the National Surgical Adjuvant Breast and Bowel Project (NSABP B-04) trial, the need for completion axillary treatment for clinically node negative patients has been questioned. This trial revealed that omitting ALND in clinically node negative patients did not affect disease-free survival (DFS) and overall survival (OS) [8]. Patients were randomized for mastectomy-only, mastectomy with ALND or mastectomy with axillary radiotherapy (RT). About 40% of the patients who underwent mastectomy-only had lymph node metastases that were not removed at the time of initial surgery. During follow-up, ipsilateral lymph nodes became clinically apparent in less than half of these patients (18.6%). Nevertheless, omitting ALND in clinically node negative patients did not affect DFS and OS, even after 25 years of follow-up and without adjuvant RT or systemic therapy.

The more recent American College of Surgeons Oncology Group (ACOSOG) Z0011 and International Breast Cancer Study Group (IBCSG) 23–01 trials investigated whether completion ALND can be safely omitted in patients with a metastasis in the SLN. The ACOSOG Z0011 trial included patients with 1–2 macrometastatic SLN(s) who were treated with breast conserving therapy (BCT) [9]. The IBCSG 23–01 trial only included patients with a micrometastasis in the SLN, but gave no restriction on type of breast surgery [10]. Most patients were treated with adjuvant systemic treatment (both 97%). Patients in these trials were randomized to completion ALND or watchful waiting. Additional lymph node metastases beyond the SLN were detected in 27% (ACOSOG Z0011) and 11% (IBCSG 23–01) in the ALND groups [9, 10]. Despite the fact that nodal metastases remained in situ in a considerable percentage of patients in the ‘watchful waiting’ groups, omitting completion ALND did not result in inferior regional recurrence (RR) rates, DFS and OS after 5-years of follow-up. These studies indicated that completion ALND can be safely omitted in presence of positive SLN(s) in patients treated with BCT and adjuvant systemic treatment. Since the outcome of the SLNB has no clinical consequence, the value of the SLNB itself is being questioned.

Clinically node negative status in the NSABP B-04, ACOSOG Z0011 and IBCSG 23–01 trials was based on negative physical examination of the axilla. Preoperative nodal staging with physical examination has a low accuracy, with a sensitivity of only 32% [11–14]. In the Netherlands, axillary ultrasound is part of standard preoperative axillary work-up. The sensitivity of axillary ultrasound (in combination with tissue sampling where deemed necessary) is approximately 80% [15]. Furthermore, a negative axillary ultrasound excludes the presence of four or more lymph node metastases, with a negative predictive value of 93–96% in the general breast cancer population [16–18]. Therefore, axillary ultrasound improves preoperative selection of node negative patients, as it selects patients with a more favourable tumour load and confidently excludes advanced nodal disease.

Several factors besides surgery have proven to decrease RR rates. For instance, it is assumed that biology plays an important role in dormancy of nodal metastases. Less than half of the patients with occult nodal metastases in the NSABP B-04 trial, developed clinically detectable lymph nodes during follow-up, none of these patients received adjuvant systemic or RT [8]. Adjuvant systemic therapy is known to decrease RR rates [19]. Primary systemic therapy can eradicate lymph node metastases with a reported pathologic complete response rates of 20–40% [20–23]. Lack of knowledge on the pathological lymph node status is nowadays hardly influencing systemic therapy indication [24]. Low RR rates in the ACOSOG Z0011 (and IBCSG 23–01) trial might be due to whole breast irradiation (WBI) following lumpectomy [25–28]. RT of the breast may contribute to the elimination of (occult) lymph node metastases by including part of the axilla [25]. A recent study has shown that, even with contemporary 3D radiation techniques, the SLN receives an elective radiation dose in 76% of patients [29]. Biology, adjuvant systemic and RT most likely diminish the risk that possible lymph node metastases left in situ develop into clinically detectable lymph nodes.

This randomized controlled BOOG 2013–08 trial proposes to demonstrate that the SLNB can be safely omitted in breast cancer patients with a clinically node negative T1–2 status undergoing BCT. This trial aims to decrease the number of breast cancer patients receiving an invasive axillary procedure, to decrease the axillary morbidity rate, thereby improving QoL and reducing the costs of SLNB without affecting regional control and survival.

### Main study objectives

Primary objective of this study is to investigate whether watchful waiting (i.e. no SLNB) is not inferior in terms of 5 and 10-year RR rate to the current axillary staging regimen in breast cancer patients with a clinically node negative T1–2 status undergoing BCT. Secondary objectives are distant-DFS, OS, local recurrence (LR) rate, contralateral breast cancer, number of delayed axillary treatment, adjuvant RT, QoL, axillary morbidity rate, and cost-effectiveness after 5- and 10-years of follow-up.

## Methods

### Study design

The BOOG 2013–08 is a Dutch prospective non-inferiority randomized controlled multicentre trial. Women with pathologically confirmed unilateral clinically node negative T1–2 invasive breast cancer undergoing BCT are randomized to SLNB or watchful waiting (i.e. no SLNB). Primary and secondary endpoints will be reported after 5 and 10 years of follow-up. The BOOG 2013–08 is a multicentre trial and will be performed in 35 participating centres (Table 1). This study design was based on the BOOG 2013–07 trial of the same research group [30].

**Table 1:** Participating centres BOOG 2013–08 study

	Name participating centre
1.	Alrijne hospital, Alphen aan de Rijn and Leiden
2.	Amphia hospital, Breda
3.	Antonius hospital, Sneek
4.	Bronovo hospital and Medical Centre Haaglanden, den Haag
5.	Canisius-Wilhelmina hospital, Nijmegen
6.	Catharina hospital, Eindhoven
7.	Deventer hospital, Deventer
8.	Diakonessenhuis, Utrecht
9.	Flevoziekenhuis, Almere
10.	Gelderse Vallei, Ede
11.	Gelre hospital, Apeldoorn
12.	Groene Hart hospital, Gouda
13.	Haga hospital, den Haag
14.	Isala Clinic, Zwolle
15.	Isala Diaconessenhuis, Meppel
16.	Jeroen Bosch hospital, den Bosch
17.	Laurentius hospital, Roermond
18.	Maastricht University Medical Centre, Maastricht
19.	Martini hospital, Groningen
20.	Maxima Medical Centre, Eindhoven and Veldhoven
21.	Meander Medical Centre, Amersfoort
22.	Medical Spectrum Twente, Enschede and Oldenzaal
23.	Netherlands Cancer Institute – Antoni van Leeuwenhoek, Amsterdam
24.	Noordwest ZHG, Alkmaar
25.	Radboud University Medical Centre, Nijmegen
26.	Reinier de Graaf Groep, Delft
27.	Rijnstate hospital, Arnhem
28.	Rivierenland hospital, Tiel
29.	Sint Antonius hospital, Utrecht and Nieuwegein
30.	Sint Elisabeth hospital, Tilburg
31.	Spaarne Gasthuis, Haarlem and Hoofddorp
32.	University Medical Centre Groningen, Groningen
33.	University Medical Centre Utrecht, Utrecht
34.	Zuyderland hospital Sittard and Heerlen
35.	Zuwehofpoort hospital, Woerden

### Study population

Women ≥18 years with pathologically confirmed clinically node negative T1–2 invasive breast cancer, treated with BCT (lumpectomy and WBI) are eligible for inclusion. Clinically node negative is defined as no signs of axillary lymph node metastases, consisting of a negative physical examination of the axilla and preoperative axillary ultrasound (or negative cyto−/histopathology in case of a suspicious axillary lymph node) [30].

Exclusion criteria are: metastatic disease; bilateral breast cancer; history of invasive breast cancer; previous surgical treatment or RT of the ipsilateral axilla (except surgery for superficially skin lesions, such as naevi or hidradenitis suppurativa); other prior malignancies, except successfully treated malignancies >5 years before inclusion, successfully treated basal cell and squamous cell skin cancer, and carcinoma in situ of the ipsilateral, contralateral breast or cervix; and pregnancy or lactation [30]. Primary systemic therapy and breast reconstructions are no exclusion criteria.

#### Axillary ultrasound

In the Netherlands, axillary ultrasound is standard care for preoperative nodal staging of breast cancer patients [30]. The following criteria are used to identify axillary lymph node metastases during an axillary ultrasound: cortical thickening, long to short axis ratio of <2 (i.e. round), effacement or replacement of the fatty hilum, and/or nonhilar blood flow [30]. If cortical thickening is >2.3 mm, fine-needle aspiration biopsy is performed [31]. Further, the radiologist can make a subjective assessment of cortical thickening during real-time imaging [16, 32, 33]. When suspicious lymph nodes are observed during an axillary ultrasound, fine-needle aspiration cytology or core needle biopsy is recommended. If more than one suspicious lymph node is present, the most suspicious lymph node is sampled [30].

#### Breast conserving therapy

BCT is defined as lumpectomy followed by WBI. The primary tumour size is determined during pathological assessment. Immunohistochemical (IHC) staining is used to determine the hormone receptor status and is considered positive if ≥10% of the cells stain positive. HER2neu status is determined by IHC, or in case of 2+ by Chromogenic In Situ Hybridization (CISH) or Fluorescence In Situ Hybridization (FISH) [30].

World Health Organization is used to define the histological tumour type. The modified Bloom-Richardson grading system is used to assess histological tumour grading. Multifocality is defined as foci or carcinoma separate from the primary breast tumor. Lymphovascular invasion is defined as ≥1 tumour cells in a lymphatic or vascular structure [30].

#### Sentinel lymph node biopsy

The SLN procedure is performed using technetium-99 m Nanocolloid as a radioactive tracer and blue dye for lymphatic mapping. Both are injected into breast parenchymal tissue surrounding the tumour, biopsy cavity or periareolar. The SLN is identified using the following triple technique: lymphoscintigraphy, intraoperative use of the gamma probe, and intraoperative detection of the blue lymphatic vessels. After removal of the SLN(s), palpation of the axilla is performed to identify and remove additional suspicious (non-) SLN(s) [30].

Each SLN is examined at three histological levels (500-μm intervals) as a minimal requirement for pathological assessment. Two parallel sections on each level are performed, one for haematoxylin and eosin (H&E) staining and one for IHC staining [30]. When H&E staining is negative IHC staining is done. Lymph nodes marked by the surgeon as non-SLNs are also examined with H&E and if negative with cytokeratin IHC staining. The diameter of each metastasis and the presence of extranodal growth must be determined. Isolated tumour cells (<0.2 mm) are considered as SLN negative [30].

### Radiation therapy

#### Dose and fractionation for whole breast radiation

A fractionation scheme equivalent to 25 × 2 Gray (Gy), 5 fractions per week is applied. Most Dutch RT centers use a scheme of 15–16 × 2.67 Gy, 5 fractions per week. In case of focal irradical resection, a higher boost dose is recommended (equivalent to 10–13 × 2 Gy) [30]. Partial breast irradiation is not allowed.

#### Delineation of whole breast radiation

The European Society for Radiotherapy and Oncology (ESTRO) guidelines of Offersen et al. are used to perform delineation of target volumes and organs at risk [34]. Delineation of tumour bed, including a clinical target volume (CTV) and a planning target volume (PTV) is obligatory. In addition, delineation of axillary nodal regions, axilla level 1, 2, Rotter nodes and 3 is obligatory, even when there is no indication for axillary radiation. In case of left-sided breast cancer, delineation of the heart and lungs is obligatory [30].

#### Radiation technique and dose distribution

The dose in the target volume (whole breast, with or without axillary and periclavicular nodes irradiation) must be between 95%–107% of the prescribed dose [30]. The Central Lung Distance must be <3 cm (in case of tangential fields) and mean lung dose should be <7.5 Gy. The Maximum Heart Distance (in case of tangential fields) must be <1 cm, and the heart volume receiving >10 Gy should be <15%. If lung or heart constraints cannot be met, some underdose in the breast can be accepted to reach the constraints, provided that the PTV of the tumour bed is adequately covered. Breath holding techniques to reduce heart dose are highly recommended for left sided breast cancer patients. For evaluation purposes, the minimum, maximum and mean dose of the axilla level 1, 2, Rotter nodes, and 3 must be recorded [30].

### Consent and randomization

Eligible patients will be informed about the study aim, randomization procedure, consequences of participating (i.e. possible adverse events), and their rights and responsibilities by the attending surgeon. Written informed consent must be obtained and randomization will be performed preoperatively. Patients will be randomized between SLNB (control arm) and no SLNB (study arm).

Patients will be stratified by: clinical tumour size (<3 cm vs. ≥3 cm), grading (grade I-II vs. III), oestrogen receptor status (positive vs. negative), HER2neu status (positive vs. negative), age (≤50, 50 ≤ 75, >75 years), primary systemic therapy (yes vs. no) and participating centre.

### Systemic therapy

According to the Dutch breast cancer guideline and multidisciplinary approach, indication for systemic therapy is determined for each individual patient [30, 31]. Adjuvant! Online can be used to estimate the 10-years breast cancer specific survival and the risk reduction by systemic therapy, with or without knowledge of the pathological nodal status. Validated gene expression profiling can be used as an addition to clinicopathologic characteristics, in case of doubt about the indication for adjuvant systemic therapy based on the traditional prognostic factors. Primary systemic therapy is allowed, if the patient has a clinically node negative T1–2 status that is amenable to BCT surgery pre-systemic therapy.

### Follow-up

The first five years of follow-up consists of outpatient clinic visits once yearly, including a physical examination of the axilla and a full-field digital mammography (FFDM). Year six to ten of follow-up, consists of a FFDM annually in patients aged ≤60 years or once every two years in patients aged >60 years [30]. Additional diagnostic imaging is only performed on indication. Axillary ultrasound is performed, in case of a clinical suspicion of axillary lymph node metastases. Staging for distant metastatic disease is performed, if an axillary lymph node metastasis is confirmed (cyto−/histopathology) or in case of a clinical suspicion of distant metastatic disease [30].

### Quality of life

A Dutch version of two validated QoL questionnaires of the European Organization for the Research and Treatment of Cancer (EORTC QLQ-C30 and QLQ-BR 23) are used to assess the QoL of breast cancer patients. To assess the subjective morbidity the validated Lymphedema Functioning, Disability and Health questionnaire (Lymph-ICF) is used. A validated short version of the Spielberger State-Trait Anxiety Inventory (STAI-trait) and Neuroticism Extraversion Openness Five Factor Inventory (NEO-FFI) is used to measure if anxiety and personality traits influences the outcome of QoL [35–38]. The combination of these questionnaires will provide information on the general and breast cancer specific QoL, subjective morbidity, and anxiety and personality traits that might influence the outcome of QoL [39]. The first QoL questionnaires are provided pre-randomisation for baseline measurement, and the following are provided post-randomization at six months, and at 1, 2, 3, 5 and 10 years. Patients are eligible for evaluation when at least the pre-randomisation questionnaire and the subsequent questionnaire are completed [30].

### Adverse events

Any undesirable experience during the study, whether or not considered related to the protocol treatment is defined as an adverse events (AEs), including seroma, postoperative haemorrhage, wound complication/infection, lymphedema of the arm or chest wall, neuralgia, paraesthesia, decreased arm or shoulder motion, muscle weakness of the arm or shoulder, and pain in the arm or shoulder. NCI/CTCAE 4.0 grading criteria is used to grade the severity of the AE into mild, moderate, or severe, in combination with the degree of limitation in activities of daily living [30].

An untoward medical occurrence or effect related to protocol treatment that results in death, hospitalisation or prolongation of existing inpatients hospitalisation, or surgery is defined as a serious adverse event (SAE). Protocol treatment is defined as BCT of the primary tumour, WBI, SLNB or completion axillary treatment. Any other operation or adjuvant treatment is not considered protocol treatment [30].

The local investigator of the participating centre is responsible for reporting the SAE to the central data centre within 24 h. The principal investigators of the study are responsible for SAE assessment and reporting to the accredited medical ethics committee within 15 days. For fatal or life threatening cases, the term is maximal 7 days for a preliminary report with another 8 days to completion the report. All SAEs will be followed until they have abated, or until a stable situation has been reached. Depending on the event, follow-up may require additional tests or medical procedures as indicated and/or referral to the general physician or a medical specialist [30].

### Statistics

#### Endpoints

Primary endpoint of this study is RR rate after 5 and 10-years of follow-up. Secondary endpoints are regional recurrence free survival (RRFS), number of delayed axillary treatment, distant-DFS, OS, LR rate, other-RR rate, contralateral breast cancer rate, diagnosis of recurrence outside the axillary region, percentage difference in the administration of postoperative RT, axillary morbidity rate, QoL and cost-effectiveness after 5- and 10-years of follow-up. RR, other-RR, LR and distant recurrence are defined according the Maastricht Delphi Consensus on Event Definition by Moossdorff et al. [40, 41]. Pathological confirmation of a RR is mandatory. All suspected lesions for recurrence on imaging, which are not accessible for histology or cytology, are presented to the Data Safety Monitoring Board (DSMB) for an independent review.

Time to event endpoints are defined as the time interval between the date of randomization and the date of first suspicion of the predefined recurrence, or the date of death, whichever comes first, measured in days. Patients in whom recurrence is not observed and are still alive are censored at the date of last follow-up. Death from breast cancer and its treatment, death from a second primary invasive non-breast cancer, and death from other- or an unknown cause are recorded [30].

Administration of (neo) adjuvant systemic therapy is registered and the percentage difference between both study arms is recorded. Axillary morbidity rate is assessed using a validated questionnaire and by predefined AE that are recorded by the treating physician. QoL is assessed using validated questionnaires. Cost-effectiveness is assessed using the EQ-5D health questionnaire [30].

#### Sample size

Previous data indicate a 5-year regional recurrence free survival rate of 99% for the control arm and 96% for the study arm. The expected regional recurrence free survival rate and non-inferiority limit of 5% (delta) with a probability of 0.8, result in a sample size of 747 per arm. When taking in account a lost to follow-up rate of 10%, 1.644 patients need to be randomized. An annual accrual of 856 patients can be achieved, when taking into consideration the incidence of women diagnosed with invasive breast cancer in the Netherlands, the rate of patients that is primarily operated (when excluding patients treated systemic therapy only, or patients with metastatic disease and frail elderly), treatment with BCT, the 35 participating hospitals, and an expected accrual rate of 30%. Therefore, two years will suffice to include the 1.644 patients.

#### Data safety monitoring board

An independent DSMB is established comprising an independent surgeon, medical oncologist, radiation oncologist and a statistician [30]. The independent DSMB will meet annually to discuss RR, other events, occurrence of AEs, and the percentage difference in administration of adjuvant systemic therapy between both study arms [30]. The DSMB can decide to alter the frequency of discussion. All suspected lesions for recurrence on imaging, which are not accessible for histology or cytology, are presented to the Data Safety Monitoring Board (DSMB) for an independent review. An interim analysis is performed by a statistician, results will be presented to the DSMB for further interpretation. The principal investigators will receive the DSMB recommendations. Should the principle investigators decide not to fully implement the DSMB recommendations, then the principle investigators have to send the recommendation to the accredited medical ethics committee, including a note to substantiate why (part of) this recommendation will not be followed [30].

#### Stopping rule

The principle investigators reserved the right to discontinue the study prior to inclusion of the intended number of subjects, but intends only to exercise this right for valid scientific or administrative reasons such as; a negative advice for continuing the study by the DSMB; in case of a percentage difference in the administration of adjuvant systemic therapy of more than 5% between both study arms; or disappointing accrual so that the total enrolment of 1644 patients seems not feasible [30].

#### Final analysis

Per protocol and in the intention to treat population will be used to analyse the primary and secondary endpoints after 5 and 10 years of follow-up. To evaluate the null hypothesis, uncorrected chi-squared statistics will be used. In case of censored data, chi-square test will be based on the Kaplan-Meier estimator. Cox proportional hazards models and Kaplan Meier estimates will be used to analyse the outcome of both groups and to assess the univariable and multivariable association between prognostic variables, treatment and events, using stratification factors. All statistical tests are 1-sided and a *p* value of 0.05 or less is considered statistically significant [30].

## Discussion

The BOOG 2013-08 trial is a Dutch prospective non-inferiority randomized multicenter trial invesitgating whether the sentinel lymph node biopsy can be safely omitted in clincially node negative breast cancer patients treated with breast conserving therpay. If the sentinel lymph node biopsy can be safely omitted in clinically node negative breast cancer patients undergoing breast conserving therapy, this study will costeffectively lead to a decreased axillary morbidity rate and thereby improved quality of life with non-inferior regional control, distant-disease free survival and overall survival.

## Abbreviations

AE: Adverse event
ALND: Axillary lymph node dissection
BCT: Breast conserving therapy
CISH: Chromogenic In Situ Hybridization
CTV: Clinical target volume
DFS: Disease-free survival
DSMB: Data safety monitoring board
EORTC QLQ-B23: European organization for the research and treatment of cancer quality of life questionnaire - breast cancer module
EORTC QLQ-C30: European organization for the research and treatment of cancer quality of life questionnaire
FFDM: Full field digital mammography
FISH: Fluorescence in situ hybridization
Gy: Gray
H&E: Haematoxylin and eosin
IHC: Immunohistochemistry
LR: Local recurrence
Lymph-ICF: Lymphedema functioning, disability and health questionnaire
NEO-FFI: Neuroticism extraversion openness five factor inventory
OS: Overall survival
PTV: Planning target volume
QoL: Quality of life
RR: Regional recurrence
RRFS: Regional recurrence free survival
RT: Radiotherapy
SAE: Serious adverse event
SLN: Sentinel lymph node
SLNB: Sentinel lymph node biopsy
STAI-trait: State-trait anxiety inventory
WBI: Whole breast irradiation
